# Preoperative Assessment of Geriatric Patients Undergoing Elective Intracranial Surgery

**DOI:** 10.7759/cureus.12284

**Published:** 2020-12-25

**Authors:** Abdullah Naji, Gabriel B Stolin, Abdelwahab Ahmed, Jason Gatling

**Affiliations:** 1 Anesthesiology, College of Osteopathic Medicine of the Pacific, Western University of Health Sciences, Pomona, USA; 2 Anesthesiology, Wayne State University, Detroit, USA; 3 Anesthesiology, Loma Linda University Medical Center, Loma Linda, USA

**Keywords:** preoperative assessment, geriatric anesthesia, elective intracranial surgery, preoperative, neurosurgery

## Abstract

A comprehensive preoperative assessment of elderly patients undergoing intracranial surgeries can reduce perioperative morbidity and mortality. Elderly patients often present with a wide array of comorbid medical conditions and extensive medication lists, which may influence the preoperative evaluation, anesthetic plan, and perioperative care. This article provides a basic overview of the preoperative assessment of elderly patients undergoing intracranial surgeries.

## Introduction and background

As the increase in life expectancy and our ability to detect new pathology grows, physicians today are operating on a higher proportion of neurosurgical elderly patients as compared to years before [1]. Over the past 25 years, there has been a significant and progressive increase in elderly patients undergoing elective intracranial surgeries [1]. The growing trend of more elderly patients undergoing elective intracranial surgeries highlights the importance of conducting a comprehensive preoperative assessment of elderly neurosurgical patients to reduce perioperative morbidity and mortality. Recent literature shows craniotomies are not as dangerous in the elderly population as previously believed [2]. Regardless, elective surgeries inherently have risks, and elderly patients tend to have a higher number of comorbid conditions. A comprehensive preoperative evaluation is of great importance for patient safety. Preoperative evaluations provide a careful assessment of the patient’s overall health and perioperative risks and are used to determine if further efforts - treatments, consultations, labs, imaging - are needed prior to surgery to optimize the patient’s health in order to reduce their risk of perioperative morbidity and mortality. There are also social aspects to consider, such as providing patient care after the surgery, which need to be discussed with the patient and their families preoperatively. Many elderly patients have more intensive needs that may require an extended rehab period with the need for a skilled nursing facility. This article will cover the general preoperative assessment of elderly patients undergoing intracranial surgeries.

## Review

Preoperative assessment

History and Medical Conditions

In addition to routine preoperative assessment, a comprehensive medical history should be elicited from elderly patients undergoing elective intracranial surgeries in order to identify any medical comorbidities that may alter preoperative, intraoperative, and postoperative management. Obtaining a complete medical history from some elderly neurosurgical patients may be difficult. In such situations, pertinent medical history information should be obtained from medical records, family members, caregivers, and the patient's other healthcare providers. Preoperative review of past surgical and anesthesia notes should be completed, as it can provide valuable information regarding future complications and help guide plans to alter anesthetic care to reduce perioperative risks.

Preoperative assessment should include specific neurological history questions that may provide important information for preoperative planning and predicting perioperative risks. Obtaining a history regarding the patient's neurological conditions is essential, and it should involve obtaining information regarding the type of lesion, time of occurrence, location, symptoms, and current treatment modalities. Questions that will help provide further information regarding the patient's neurological conditions include inquiries regarding the history of stroke, transient ischemic attacks (TIA), seizures, and signs and symptoms of increased intracranial pressure (ICP).

Cardiovascular comorbidities must be identified and optimized before neurosurgery. The Revised Cardiac Risk Index for Pre-Operative Risk (RCRI) can be utilized to aid in risk-stratifying patients, which helps to provide guidance in understanding when to use specific preoperative labs (serum BNP) and postoperative cardiac monitoring (EKG, troponins). Furthermore, RCRI aids patients in understanding their individualized risk prior to undergoing surgery, which is of utmost importance in the discussion of informed consent. The decision for specific preoperative evaluations and interventions must follow current guidelines [3]. The current American College of Cardiology/American Heart Association (ACC/AHA) guidelines advise that perioperative beta-blockers should be utilized in patients who are chronic beta-blocker users and patients with intermediate-risk or high-risk myocardial ischemia noted by preoperative stress testing [3]. Furthermore, ACC/AHA advocates that it is reasonable to initiate beta-blockers before surgery in patients with three or more RCRI risk factors (e.g. heart failure, diabetes mellitus, coronary artery disease, renal insufficiency, cerebrovascular accident) [3-4]. It also emphasizes that beta-blockers should not be started on the day of surgery. Statins are effective in preventing cardiac events and should be continued in patients currently taking statins who are scheduled for non-cardiac surgery [3,5-6]. Elderly patients with chronic hypertension have increased cerebrovascular resistance resulting in the requirement for higher mean arterial pressure (MAP) to maintain cerebral profusion pressure (CPP). This is an important consideration since such patients will have increased susceptibility and poor tolerance to cerebral hypoperfusion in acute hypotensive perioperative events. Lastly, current ACC/AHA guidelines advocate waiting four to six weeks after a myocardial infarction (MI) prior to performing elective surgery [7].

A review of the respiratory system is vital to ensure essential considerations are taken during airway placement, intubation, oxygenation, and ventilation of the patient. Awareness, evaluation, and understanding of an elderly neurosurgical patient’s respiratory comorbidities are significant since efforts can be taken to stabilize their disease preoperatively and ensure sufficient oxygenation and ventilation intraoperatively and postoperatively. Elderly neurosurgical patients are at risk of respiratory complications, including gastric aspiration resulting in pneumonitis and pneumonia, which can contribute to adverse postoperative neurological outcomes [8]. Patients must be preoperatively assessed for a history of obstructive sleep apnea (OSA) since it increases the incidence of perioperative complications, and thus influences the anesthetic surgical planning [9]. The STOP-BANG questionnaire is a concise and valid screening tool to be utilized preoperatively to identify patients with high-risk OSA [9].

Diabetes mellitus (DM) influences the anesthetic surgical planning and must be preoperatively optimized. Hyperglycemia is associated with poor wound healing and an increased risk of infection. Hyperglycemia negatively impacts the outcomes of neurosurgical patients [10]. In a prospective analysis study, the preoperative initiation of tight glycemic control reduced the mortality rate in neurosurgical patients [11]. Metformin should be discontinued 24-48 hours before surgery to reduce the risk of lactic acidosis. Preoperatively, glucose levels must be frequently monitored, and an insulin sliding-scale should be utilized to obtain blood glucose levels between 100 mg/dL and 150 mg/dL.

Renal, hepatic, and bleeding disorders all can have implications on surgical anesthetic planning and must be evaluated. Contrast dye, intravascular volume depletion, mannitol, aminoglycoside antibiotics, nonsteroidal anti-inflammatory drugs (NSAIDs), angiotensin-converting enzyme inhibitors (ACEI), and angiotensin receptor blockers (ARB) increase the risk of renal failure and should be avoided or minimized.

A comprehensive preoperative history involves inquiring about the patient’s allergies, medications, and prior surgical history. Patients should also be questioned about past history of anesthetic complications including difficult airway, intravenous (IV) access difficulty, postoperative pain, and family history of anesthesia complications. Physicians should also inquire about the patient’s alcohol and smoking history and encourage preoperative cessation. Cigarette smoking cessation should occur between six to eight weeks prior to surgery and has been associated with improved perioperative morbidity [12].

Medications

The patient’s current medications and allergies must be reviewed. Most medications are continued preoperatively for elderly neurosurgical patients. Chronic pain medications in the elderly are continued preoperatively since they control postoperative pain and blood pressure [13]. The majority of antihypertensive and antiarrhythmic medications are continued preoperatively to prevent sudden-onset preoperative rebound hypertension, with the exception of ACEI and angiotensin receptor blockers (ARB), which are discontinued preoperatively. ACEI and ARB are associated with an increased risk of refractory intraoperative hypotension, hence it is recommended that patients discontinue these medications on the day of surgery [14-15]. Patients must stop utilizing oral hypoglycemic medications before surgery. Specifically, metformin should be discontinued at a minimum of one day before surgery and restarted two to three days postoperatively after renal tests demonstrate normal renal functioning since metformin is associated with lactic acidosis [13,16]. Sliding-scale insulin should be used to optimize perioperative glycemic levels.

The management of preoperative anticoagulation cessation in elderly neurosurgical patients is complex since such patients have many comorbidities (atrial fibrillation, mechanical valves) that increase their risk of thromboembolism. At the same time, neurosurgical procedures are invasive and are associated with increased bleeding risks. Therefore, a balance must be achieved between the patient’s risk for thromboembolism and preventing excessive bleeding when deciding the preoperative discontinuation duration of anticoagulant and antiplatelet medications. For elderly neurosurgical patients, warfarin should be stopped five to six days preoperatively [17-18]. Further consultation is needed when the risk of thromboembolism is higher in patients with atrial fibrillation, systemic embolism, and mechanical valves. In these individuals, the warfarin cessation time should be minimized, and bridging therapy with heparin should be initiated preoperatively and stopped a few hours prior to surgery [18].

New oral anticoagulants (NOAC) are gradually replacing warfarin in clinical practice. NOAC now have a wide array of indications, including their use to prevent stroke in non-valvular atrial fibrillation, prevention of venous thromboembolism (VTE) and pulmonary embolism (PE), treatment of VTE and PE, and as thromboprophylaxis in certain orthopedic surgeries [19]. Therefore, in an aging population with complicated comorbidities and indications of NOAC increases, these medications will be frequently encountered by anesthesiologists caring for elderly patients undergoing neurosurgery. The duration of NOAC discontinuation prior to surgery depends on the bleeding risk of elective surgery. Intracranial surgeries are considered high risk, and the current recommendation advocates that NOAC are discontinued three days prior to high bleeding risk surgeries [20]. Elderly neurosurgical patients are considered a high-risk bleeding group. Thus it is generally recommended that their NOAC is restarted two to three days postoperatively, provided there are no signs and symptoms of hemostasis abnormalities or neurosurgical contraindications [20-21].

Elective intracranial surgery in the elderly poses a high bleeding risk with moderate to high thrombotic risks. Antiplatelet medications are widely used by the elderly for the secondary prevention of stroke. Aspirin should be discontinued two to three days before the elective major neurosurgical procedure [13]. Clopidogrel should be discontinued at least five to 10 days prior to major neurological surgery when the risk of ischemia is low [22].

Physical Examinations

The preoperative physical examination must focus on the highest risk areas for the elderly patient population to ensure proper surgical risk stratification. Preoperative physical examination of elderly neurosurgical patients should focus on assessing the patient's airway, cardiac, respiratory, and neurological systems.

For anesthesiologists, assessing the airway is one of the most essential physical examinations to perform [23]. All patients must be evaluated for difficult airways. A comprehensive airway examination should be completed in accordance with standard evaluation guidelines [24]. Some elderly patients undergoing elective intracranial surgeries may have restricted mouth opening or limited neck mobility due to a variety of reasons, including cervical spine disease, acromegaly, high intracranial pressure, or old-age limitations. In such patients, performing the upper lip bite test is a quick and accurate method to evaluate the airway. Studies have demonstrated that the upper lip bite test has a higher specificity for predicting difficult airway when compared to the thyromental distance and inter-incisor space test, underscoring its usefulness in elderly patients with restricted neck mobility and mouth opening [25].

Establishing baseline cardiac function is key for risk stratification in elderly patients [26]. A physician must elicit a complete history of cardiac abnormalities such as aortic stenosis, myocardial infarction, and atrial fibrillation. Cardiac physical examination should assess the patient's heart rate, rhythm, pulses, presence of carotid bruits, heart murmurs, and signs of cardiac failure. Preoperative baseline blood pressure and heart rate measurements should be obtained, as they will aid the anesthesiologist regarding appropriate blood pressure control during the surgery [27]. Proper oxygenation and ventilation at baseline is an important prerequisite to elective surgery. The first step to establishing an individual's respiratory function is to assess breath sounds bilaterally for wheezing, rales, rhonchi, or stridor. Identifying early signs of airway disease that could complicate anesthetic management is key to a successful case. Preoperative oxygen saturation is also used to establish the patient's baseline respiratory function.

A comprehensive preoperative neurological examination assessing cranial nerves, sensory and motor function, and cerebellar function is required to establish baseline neurological status. Preoperative neurological examination should include an evaluation of cognition, which can be gained by communicating with the patient or asking family members regarding the patient's normal state of cognition. Depending on the past workup, it is useful to use previous exams, such as multi-mini mental state exams, as a baseline. Furthermore, any nervous system deficits found in the neurological exam must be carefully noted to compare postoperatively. When operating on the brain, many unexpected events may occur. Proper documentation is required to note a new deficiency postoperatively.

Preoperative Investigations

The current American Society of Anesthesiologists (ASA) Practice Advisory for pre-anesthesia evaluation recommends that specific preoperative investigation tests should be done based on the patient's comorbid medical conditions and the type of surgery the patient is receiving [28]. Elderly patients undergoing elective intracranial surgeries may experience blood loss. Preoperative baseline complete blood count (CBC) labs must be obtained and the patient should be cross-matched for possible intraoperative blood transfusion. Severe intraoperative anemia of elderly neurosurgical patients can increase the risk of tissue hypoxia due to reduced oxygen delivery. Hematocrit levels of 30%-33% in elderly neurosurgical patients ensures an optimal oxygen-carrying capacity that minimizes the risk of tissue hypoxia [29]. Other laboratory tests, including serum electrolytes, chemistry profile, glucose, renal function tests, and coagulation parameters, should be obtained preoperatively. These laboratory tests are usually acceptable up to six monthly preoperatively given the patient has not experienced changes in their medical history or had significant clinical events [30].

Preoperative electrocardiogram (ECG) should be conducted in elderly patients with a medical history of cardiovascular disease, respiratory disease, or multiple cardiovascular risk factors [28]. Preoperative chest radiography should be considered for elderly patients with clinical characteristics, including a history of chronic obstructive pulmonary disease (COPD), recent upper respiratory infection, cardiac disease, or history of smoking [28]. Further cardiac or respiratory preoperative evaluations should be guided by the patient's clinical presentation, preoperative medical history, physical examination, results of previous preoperative investigations, and type of intracranial surgery.

Preoperative neurological testing should be conducted based on the patient's clinical presentation and history of previous neurological testing. For example, elderly neurosurgical patients with a history of TIA or stroke should be screened with a Doppler ultrasound or computed tomography angiography (CTA); especially if such imaging evaluations have not been previously done or the patient's clinical presentation has worsened since the date of initial imaging [31].

Communication

One of the most critical aspects of the preoperative assessment is to ensure agreement from all parties: the patients and their families, the surgeons, and the anesthesia team. Proper communication is a complex topic, especially in elective surgeries. The benefits and risks of the procedure, anesthesia, and appropriate follow-up and postoperative care must be completely explained to the patients and their families to make an informed decision. As these are elective surgeries, we must also educate on what can happen if the patient declines surgery. Some pathology might not adequately advance to require surgery, such as meningioma or vestibular schwannomas. In these circumstances, rather than putting patients through a craniotomy, simple observation with serial imaging is an option to consider.

Another situation that is pervasive in the elderly patient population that must be evaluated is anticoagulation. Many elderly patients are on anticoagulation therapy for various reasons, including mechanical heart valves or atrial fibrillations. Many cannot safely discontinue anticoagulation for long periods of time. Alternative options such as stereotactic radiosurgery (SRS) for small tumors in the brain have been shown to be effective [32]. For discrete surgical lesions, such as deep brain stimulation (DBS) for essential tremor or Parkinson’s, the use of Gamma Knife or magnetic resonance imaging (MRI)-guided focused ultrasound therapy may be considered. In some cases, there is a higher risk when performing bilateral surgery when compared to DBS. Alternative options are more suitable for unilateral pathology.

Some patients with brain tumors may have an altered level of consciousness that may affect their ability to consent. In these cases, transparent discussions with the patient’s family (the individual with the power of attorney) are vital to ensure the patient is able to follow preoperative instructions and follow-up care. Lastly, the anesthesiologist and surgeon must agree and must use high-level communication before, during, and after the operation.

## Conclusions

Elective intracranial surgery in the elderly necessitates systematic preoperative assessment by an anesthesiologist. When operating on the brain, small alterations in physiology can significantly change the amount of care needed. Such considerations are important in the elderly population, who may be sicker and have more comorbidities than younger patients. Successful surgery is dependent on proper preparation and appropriate preoperative assessment. Clear communication and expectations between the patient, surgeon, and anesthesiologist will give the operation the best chance of success.
